# Learning strategies of general practitioners striving to achieve point-of-care ultrasound competence: a qualitative study

**DOI:** 10.1080/02813432.2022.2036483

**Published:** 2022-02-15

**Authors:** Camilla Aakjaer Andersen, Marie Espersen, John Brodersen, Janus Laust Thomsen, Martin Bach Jensen, Annette Sofie Davidsen

**Affiliations:** a Center for General Practice at Aalborg University, Aalborg, Denmark; b Department of Public Health, Faculty of Health Sciences, Research Unit for General Practice and Section of General Practice, University of Copenhagen, Copenhagen, Denmark; c Primary Health Care Research Unit, Region Zealand, Denmark

**Keywords:** Diagnostic methods, education, family medicine, general practice, qualitative research, ultrasonography

## Abstract

**Background:**

Point-of-care ultrasonography (POCUS) is increasingly used in general practice despite the lack of official educational programmes or guidelines for general practitioners (GPs).

**Aim:**

To explore how GPs have learnt to use POCUS and which barriers they have encountered in their learning process.

**Design and setting:**

Qualitative study conducted in office-based general practice in Denmark.

**Methods:**

Semi-structured interviews were conducted with 13 GPs who had implemented POCUS without supporting guidelines or regulations. Analysis was carried out using systematic text condensation. The interview data for this study were collected along with data used in a previous study.

**Results:**

The participating GPs described having composed their own ultrasound education following a continuous learning process. Basic POCUS competences were achieved through formalized training sessions at hospital departments or courses. The GPs further developed and expanded their scanning skills through additional courses and continuous self-study practice on patients often while consulting internet sources, textbooks or colleagues. Lack of available ultrasound courses, supervision, and clinical guidelines together with time constraints and financial aspects were mentioned as barriers to their ultrasound training.

**Conclusion:**

This study showed how GPs had composed their own ultrasound education individually and differently, guided by their own experiences and beliefs about good clinical practice. Formalized ultrasound training was considered a prerequisite for achieving basic ultrasound competences while continuous practice was considered paramount to develop and maintain scanning skills. There were several obstacles to overcome in the learning process including lack of supervision, guidance, and opportunity for practicing skills.

**Key points**
- Little is known about the educational needs of general practitioners striving to achieve ultrasound competences.
- General practitioners described using formalized training to achieve basic scanning competences and continuous self-study and practice to further develop their skills.
- Lack of time, supervision, clinical guidelines and ultrasound courses were considered barriers in the learning process together with financial aspects.

## Introduction

Point-of-care ultrasound (POCUS) is increasingly used by clinicians at the frontline to aid diagnosis and guide clinical procedures [1–3]. POCUS has been introduced into the curriculum at medical schools [4] and residency programmes [5–7]. Some medical specialties have developed POCUS guidelines and educational programme recommendations [1,8]. Though POCUS is also used in primary care [9–11] there is generally a lack of formal training programmes and educational recommendations for general practitioners (GPs) [12,13].

POCUS is an abbreviated and focused ultrasound examination different from the traditional comprehensive ultrasound examination performed by an imaging specialist [2]. Correspondingly, educational programmes for secondary care specialists cannot necessarily be transferred to and applied in training programmes for GPs. The working conditions in office-based general practice may not offer similar possibilities for training and supervision [14] and practicing scanning skills is challenged by workload and time frames in the general practice consultation [15]. Furthermore, the low pre-test probability of disease in general practice and the rare encounter of severe disease may challenge the GPs’ ability to recognize pathology [16].

A systematic review [13] concerning training programmes for GPs found that the programmes were largely adapted from secondary care and varied considerably in amount of training and educational elements. Little attention has been given to explore the educational needs of office-based GPs or experiences of GPs, who had adopted POCUS without support or guidelines.

Therefore, this study aimed to explore how GPs had learnt to use POCUS and which barriers they encountered in their learning processes.

## Methods

Between August 2016 and February 2017, individual semi-structured interviews [17] were conducted with Danish GPs who used POCUS in their daily work. The interviews aimed to gain insights into the GPs’ experiences of using POCUS and their process of learning to use it. Individual interviews gave the GPs the opportunity to share their experiences and account for their behaviour and decisions without having concerns about their colleagues’ opinions, judgment or critique. The interviews were designed to cover several research questions related to the use of POCUS in general practice. In a previous publication [18], we used parts of the interviews to report findings related to the use of POCUS in the general practice consultation. Using the same interviews as the data source, we used other parts for a separate analysis conducted to answer a different research question.

The research team consisted of a GP resident (CAA), a medical student (ME), and four GPs (JB, JLT, MBJ and ASD). Four of the researchers had experience in conducting qualitative research (CAA, JB, JLT and ASD) and two had experience in using POCUS in general practice (CAA and MBJ).

### Setting

The study was conducted in general practice in Denmark where approximately 3400 GPs work in 1800 practice units [19]. Danish GPs are self-employed, but with a collective agreement with the Danish Regions. Patient treatment is tax financed and free of charge for patients. GPs are paid by a mixture of capitation payment and fee-for-service [14]. However, there is no fee for performing POCUS and POCUS was only performed by around 4% of Danish GPs in 2016 [9]; however, from our thorough knowledge in the area of POCUS in Danish general practice, we assume that around one in ten perform POCUS in 2021.

### Ethics

The participating GPs provided informed consent to participate in the study. All study data were pseudo-anonymized using de-identification numbers. Only the principal investigator (CAA) knew the identity of the GPs. The study was approved by the Danish Data Protection Agency (2016-41-4768) and the Committee of Multipractice Studies (MPU-20-2016). According to Danish Law, no ethical approval was needed.

### Recruitment

GPs using ultrasonography were invited to participate in the study through teaching sessions and networks for Danish GPs using POCUS [18]. Eligible GPs were encouraged to provide their background and contact information in a small questionnaire designed for this purpose. Thirty-four POCUS-users signed up to participate. From these, we purposely selected and recruited participants stepwise aiming for maximum variation as regards age, gender, working experience as GP, experience in using POCUS, location, size and organization of the clinic. After recruiting 13 participants we reached information power [20].

### Data collection

A comprehensive interview guide was developed by CAA, MBJ, ASD and JB based on findings from a preceding literature review [12] and discussions within the research group. The interview guide covered several research questions beyond the scope of the present study (the complete interview guide is available as an additional file in reference [18]. The interview guide provided structure and suggested questions within the areas of interest whilst a flexible approach allowed for an exploration of the GPs’ narratives. CAA conducted all interviews in the GP’s practice during or immediately after normal working hours. The interviews lasted from 40 to 90 min and the GPs were financially compensated for the time used. Interviews were audio-recorded and transcribed verbatim by CAA using Soundscriber (Softpedia, University of Michigan) and Microsoft Word (Microsoft office 2016, Redmond, USA). The interviews, transcriptions and the analysis were conducted in Danish and the analytical results and quotations were later translated into English.

### Data analysis

**Figure 1.**
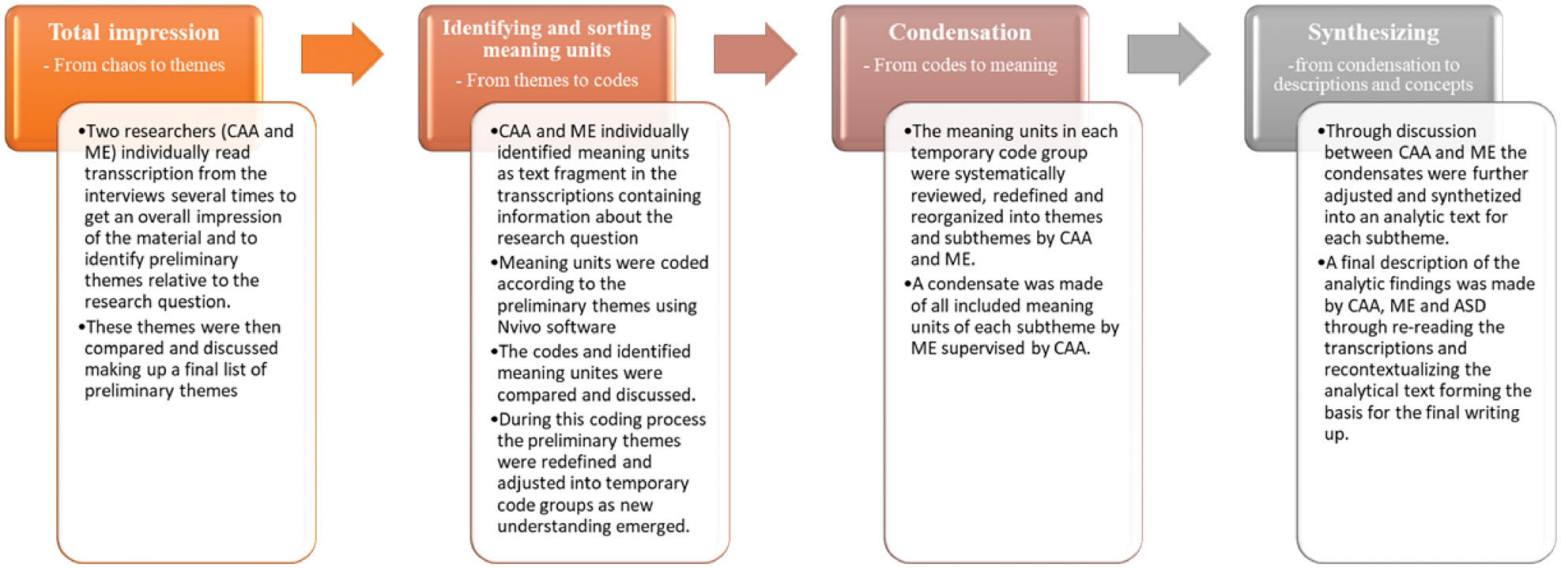
Analytical process. The analysis was conducted using systematic text condensation [26].

The transcriptions were analyzed using systematic text condensation, which is an inductive thematic cross-case analysis [21]. The analysis was conducted in four steps as elaborated in Figure 1.

## Results

Table 1 reports baseline characteristics of the included GPs. The GPs described how they had made their own decisions and assembled their ultrasound education. They said they navigated according to ethical principles and their beliefs about good clinical practice. However, they also said that they were left to draw from experiences from other medical specialties and had to find out themselves what could be integrated into their everyday work and the clinical context of general practice.

**Table 1.** Characteristics of participants.

	*N*
Gender	
Male	11
Female	2
Age, years	
40–50	6
51–60	4
61–70	3
Experience with ultrasonography	
<2 years	7
2–5 years	4
>5 years	2
Years in practice	
>20 years	3
10–20 years	6
<10 years	4
Practice location	
Urban	8
Mixed urban and rural	5
North Denmark Region	2
Central Denmark Region	4
Region of Southern Denmark	2
Region Zealand	0
Capital Region of Denmark	5
Type of practice	
Partnership practice	9
Collaboration practice	2
Solo-practice	2
Practice size	
<2000 patients	4
2000–5000 patients	4
>5000 patients	5

**Figure 2.**
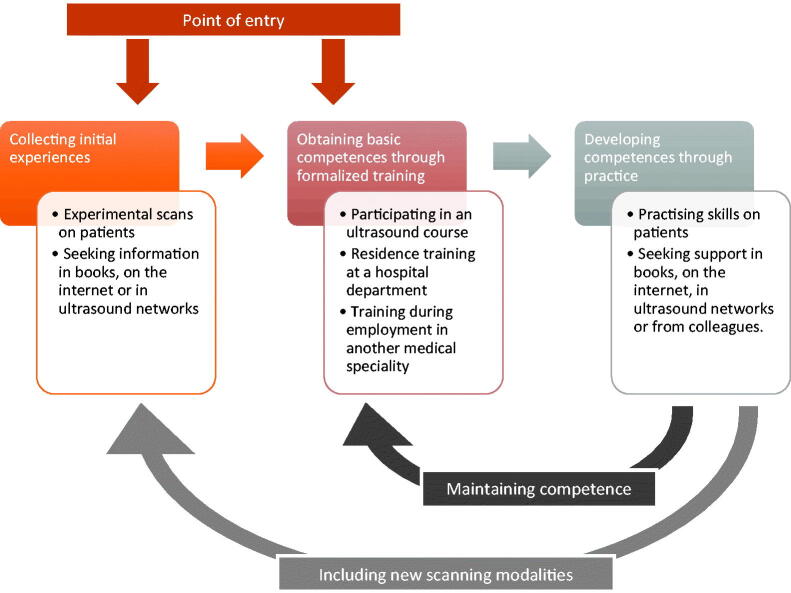
Learning process of general practitioners to obtain scanning proficiency. The general practitioners (GPs) described moving through a gradual learning process as they strived to obtain competence in performing point-of-care (POCUS) ultrasound examinations. The GPs had different starting points for learning POCUS; some had prior scanning experience, others started from scratch. Basic POCUS competences were obtained through practical training during courses or employment at hospitals. These competences were further developed through scanning on patients in general practice. Some GPs ended the learning process here. Other GPs returned to update their scanning skills on courses and most GPs started collecting new early experiences with scanning modalities aiming to obtain new competences.

The GPs described different starting points. Especially younger GPs had used ultrasound in their residence training in hospitals, whereas others started as complete novices. However, in spite of this they all moved though the same gradual learning process when striving to achieve scanning proficiency (Figure 2), but their planning and approach to learning differed considerably. Some GPs described a deliberate approach and a clear strategy while others described themselves as ‘happy amateurs’ who had plunged into using POCUS with great enthusiasm, making random choices during the learning process. The analysis revealed the following themes:

- Using collected experiences as a starting-point
- Obtaining basic ultrasound competences
- Participating in ultrasound courses
- Developing scanning competence through self-study and practice
- A continuous learning process
- Obstacles to overcome during the learning process.

### Using collected experiences as a starting-point

All the GPs described how implementing ultrasound examinations into their daily practice had required preparatory considerations. They described having gathered information about *what-to-do* and *how-to-start* from ultrasound-experienced colleagues and searches on the internet. In this process, they had found a lack of knowledge about POCUS use in general practice. They had been left to draw on experiences from available sources and the early-adapters, who had been using ultrasound for many years, described that they had been left to a trial-and-error approach.

Some participants had signed-up for an ultrasound course before they bought the ultrasound scanner, while other participants described how they had collected early experiences by simply venturing into performing ultrasound examinations on patients:

…so I bought it [ultrasound equipment] and then I just started using it on patients, where I thought it would be interesting to scan them – regardless which organ systems or which symptoms they presented – I just placed the probe [on the patient], read a bit about it and then later I participated in a course (GP2)

This experimental use of POCUS gave the GPs early experiences with different scanning modalities. However, all GPs described having realized that learning-by-doing was insufficient to obtain ultrasound competences. Some explained that this preunderstanding had guided their initial cautious approach, whereas others described having made this discovery after they started scanning patients:

I started reading a bit about it and people [patients] were willing to let me practice […] so I was using the scanner, but it occurred to me that I wasn’t good enough, so I decided to go to a basic seminar at a local hospital (GP5)

The GPs explained that these early experiences created a dawning awareness about specific difficulties and a focus on where they needed more training and knowledge.

### Obtaining basic ultrasound competences

All participating GPs talked about the importance of obtaining *basic ultrasound competences.* However, as a construct, they described basic ultrasound competences very vaguely. Several GPs mentioned the ability to produce valid ultrasound images, while no one mentioned the ability to integrate the findings into the clinical context in general practice.

All GPs described having acquired basic knowledge and skills through different kinds of formalized training: some had received a more-or-less structured introductory ultrasound training during employment at hospital departments, others had participated in ultrasound courses, and a few GPs had arranged teaching sessions with gynaecologists or imaging specialists to gain basic ultrasound competences.

The younger GPs said that ultrasound training had been part of their residency training in hospital departments where ultrasound examinations formed part of the standard examination of patients, e.g. at the gynaecological and obstetrical departments. Some GPs had more extensive experiences from longer positions at gynaecological or cardiological departments. However, these positions were more than 10 years previously. Still, they used their prior experience as a foundation for using POCUS in general practice.

I have experience from my employment at a gynaecological department in 1985–86, where we used ultrasonography. There we became skilled in the few things we used it for (GP19)

Almost all GPs said that they had updated and expanded these previously obtained competences through at least one formalized ultrasound course. Such courses were described as a fundamental part of achieving ultrasound competences, and GPs with no prior experience from a hospital position had all gained their basic ultrasound competences by participating in a formalized ultrasound course.

### Participating in ultrasound courses

The GPs described that they signed up for ultrasound courses to receive structured and formalized training with the assumption that scanning proficiency would be sufficiently obtained by participating in an established course. However, they said that they felt left alone to choose which ultrasound courses to sign-up for and that finding relevant courses was difficult. Some GPs described a wide scale search to find specific courses tailored for GPs, while others revealed a less critical approach choosing randomly among available ultrasound courses.

Although the GPs generally described good learning outcome from the courses, they also said that the courses varied greatly regarding organisation, time, scope, and content. The GPs highlighted that they participated in the courses to acquire the technical ability to produce ultrasound images and they emphasised the importance of practical hands-on training and guidance.

All courses [that I have participated in] have included hands-on training in various degrees. There have been more or less theoretical training, but all [courses] have included hands-on – in some courses up till 90% (GP3)

The GPs had participated in courses of different length and extent. Several GPs had participated in a one-year ultrasound education [16] with longitudinal feed-back on recorded scans. Others had participated in one-day practical ultrasound sessions. Some courses had included an evaluation of ultrasound competences, but most courses had not. Several GPs called for more longitudinal training and supervision and for clinical decision aids to be included in the courses. Moreover, the GPs said that they did not feel sufficiently competent after participating in the available courses, but needed to develop routines and collect scanning experiences.

### Developing scanning competence through self-study and practice

All GPs agreed that apart from ultrasound courses, practicing skills on patients was the most important way to achieve, develop and maintain competences. They explained how they sometimes performed ultrasound examinations on patients without clinical indication just to practice their skills or train their ability to distinguish between normality and pathology. The GPs also described extending their clinically indicated ultrasound examinations to include organs from which the patients had no symptoms. The GPs did this to facilitate their own learning process.

If I am for example doing an abdominal POCUS examination and I do not find anything, then I often extend the examination to other areas, just to practice. (GP2)

The need for continuous training was not limited to ultrasound novices. Some more experienced GPs described a need to practice to maintain skills. Some recounted that they exchanged experiences and discussed findings with colleagues within the clinic by inviting them to look over their shoulders while they were examining patients with POCUS. Some arranged training sessions with patients to provide opportunity and extra time to practice more difficult examinations. One GP described:

I have arranged one afternoon each week where I stay after work to do ultrasound examinations. There I have 20 minutes appointments. I tell the patients from the beginning that I have to practice and that I will treat them just as well as before. Typically, the session takes place using books and YouTube. (GP8)

The GPs described using different knowledge sources to gain further insights. They used text books, different internet sources like YouTube or ultrasound podcasts and/or reviewing saved ultrasound images. They did not search specifically for scientific evidence, although one GP described having searched the literature for evidence of abdominal aortic aneurism screening in primary care.

Yes, I heard some GPs talking about it, so I went home to study evidence for screening the aorta at the annual check-up. I found out, that it was not relevant. (GP15)

### A continuous learning process

All GPs described being in a continuous learning process with a need for more education and a crucial need to practice their ability to produce and interpret ultrasound images. Some described themselves as being in the beginning of this process, whereas others considered themselves more experienced, but as they wanted to include new scanning modalities in their repertoire they returned to collect early experiences through experimental use and scanned areas, in which they had not received formalized teaching (Figure 2). They recounted that this experimental use was motivated by a professional curiosity that had to be followed by formalised training. Some GPs explained that at a certain timepoint in the learning process they performed both POCUS examinations in which they were experienced and novices (to collect early experiences).

Many GPs described a structured process of developing skills where they started scanning just one or a few anatomical areas to become familiar with these examinations before moving on to include more examinations in their portfolio. Other GPs, however, said that they started scanning exploratively within several anatomical areas before deciding on a portfolio suited for their everyday practice. Despite having an established practice with POCUS, all GPs, except one, planned continuously to expand their portfolio or update their competences by participating in future ultrasound courses. As one of the GPs described:

We attended an ultrasound course before we bought the scanner. Then we participated in another course at the same time as we bought the scanner, and then again afterwards. I am not at all done with ultrasound courses (GP12).

Some also described a structured plan for their continuous ultrasound education involving courses and allocated time to practice their ultrasound skills. Other GPs described a desire for continuous education, but without a structured plan. The continuous education typically aimed at more advanced examinations. However, the GPs differed as regards the direction they chose to develop their ultrasound competences e.g. in orthopaedic, abdominal, gynaecological or obstetrical examinations. The direction was primarily based on their own interests.

### Obstacles to overcome during the learning process

The GPs experienced several obstacles for their learning process. Some concerned the working conditions in general practice and some a lack of guidance. Most GPs described ways to overcome these obstacles, whereas some felt these obstacles limited their learning process.

The GPs described that their great workload and tight timeframe limited the time to practice their ultrasound skills during their normal working hours and they sometimes had to abandon an ultrasound examination:

We are already racing against time. The consultation timeframe limits how much time you have to practice. (GP4).

In addition to the time constraints, the GPs narrated that only few patients had conditions suitable for a POCUS examination and as a result they only used ultrasound a few times a day. Only a few GPs had scheduled extra time for ultrasound examinations in their consultations. The extra time spent on an examination was described as *money lost* for the GP due to the lack of a fee for performing the examination. Some GPs explained that they had made organisational changes to make room for practising ultrasound examinations, for example by re-booking patients:

… the main reason for re-booking the ultrasound examination a few days later is for me to be able to prepare for the scan. (GP17)

Although the GPs described POCUS related expenses as investments in their personal job satisfaction, the majority also described financial barriers. The cost of the ultrasound device entailed that they did not have the high-end equipment that they felt would ease the learning process, and the missed income during absence from the clinic was mentioned as a barrier for taking additional courses.

Some GPs felt that the lack of formalized education and ultrasound courses targeted at general practice was a barrier. This also applied to the amount of extra training they needed to expand their curriculum, the geographical placement of ultrasound courses, and a limited number of seminar days for the individual GP. One GP explained:

I did consider participating in an ultrasound course in Copenhagen, but then I chose other seminars instead. I have a limited number of seminar days. Therefore, I have not succeeded in taking a course yet. It is clearly a barrier for me in relation to doing more ultrasound examinations. (GP4)

The limited supply of ultrasound courses targeted at general practice made GPs search for education elsewhere. Some described having participated in courses targeted at other medical specialities and in other countries, e.g. in Norway and Austria. Besides courses targeted at radiologists, some GPs described attending ultrasound courses targeted at rheumatologists, emergency doctors, and sonographers. Most of the ultrasound courses were organized by medical societies, but a few GPs had attended an ultrasound course organized by the manufacturer of their ultrasound device.

Several GPs declared that they lacked supervision of their ultrasound scans even though they were used to performing other types of examinations like ECG recordings, tympanometry or spirometry without supervision. One GP described participating in the same ultrasound course more than once to get supervision.

In January I have planned to participate in a seminar, which I have attended previously, simply because I need more supervision. (GP12)

Some GPs were very cautious and humble in their approach and called for a recommended curriculum and clinical guidelines to support their training and their ability to maintain ultrasound competences over time. They described guidelines as opportunities for self-development. Others were more adventurous and welcomed guidelines as inspiration, but feared regulations would lead to unnecessary restrictions in their use of POCUS.

## Discussion

### Summary of main findings

The participating GPs described having individually composed their own ultrasound education following the same continuous learning process. Early collected experiences were used as a starting point for learning new scanning modalities. Basic ultrasound competences were obtained through formalized training either at ultrasound courses or during hospital employments. These competences were further developed through self-study, practical training of skills on patients, consulting internet sources, textbooks or colleagues. This learning process was repeated when the GPs strived to include new scanning modalities in their portfolio. Some described a deliberate and structured approach in this learning process while others followed a more random process. Lack of available ultrasound courses, supervision, and clinical guidelines together with time constraints and financial aspects were mentioned as the main barriers to learning to perform ultrasonography.

### Strengths and limitations

To our knowledge, this study is the first to provide an in-depth description of GPs’ learning strategies for achieving and maintaining ultrasound competences. The interviews were conducted four years ago, and since then short ultrasound courses aimed at GPs have become more available. However, although following these courses GPs are still largely left on their own. Longitudinal training programs and official guidelines to support GPs in implementing POCUS are still lacking.

All informants had previous knowledge of the use of ultrasound and were pioneers in using POCUS in general practice. Hence, they may represent a minor sub-group of highly motivated GPs. This must be taken into consideration if planning a broader implementation and education of GPs.

The study was conducted in general practice in Denmark and cannot necessarily be applied to other settings. Still, the findings can probably be transferred to health care systems with no formal ultrasound education for GPs, which is the case in many countries [22].

### Findings in relation to learning theories and existing knowledge

**Figure 3.**
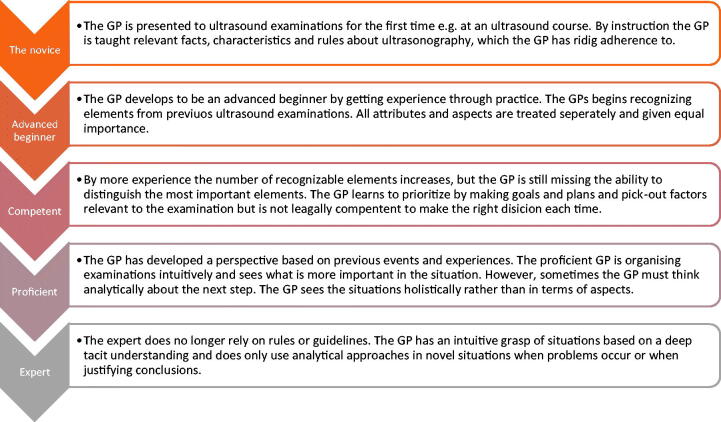
– Findings in relation to The Dreyfus and Dreyfus model. The Dreyfus and Dreyfus model [28] describes five competence levels that practitioners have to go through when they strive to learn a new competence. This figure illustrates our findings according to the model.

Training and achieving competences can be understood at an individual and an organizational level. At the individual level, the Dreyfus and Dreyfus model [23] describes practitioners’ development from novices to experts. Figure 3 illustrates our findings in relation to this model. Most participating GPs provided descriptions placing them at level 1-3 of the model. However, given the circular learning process, each GP may be at different levels as regards the different scanning modalities they have included. The Dreyfus and Dreyfus model emphasizes the importance of the practical experience and training and points to possible pitfalls at level 1-3. Hence, for skills improvement, continuous practice is paramount. Continuous supervision and feed-back on the performed scans may be required at this point in the learning process. However, it is typically not possible for GPs to obtain supervision in a general practice setting. The GPs in our study called for more supervision to be included in training programmes. In addition, lack of time to practice was mentioned as a barrier, which has also been found in hospital settings [24,25]. Even though the GPs did not consider it, the low prevalence of patients in general practice with conditions suited for POCUS examinations [11] may further challenge the opportunities for developing tacit knowledge.

**Figure 4.**
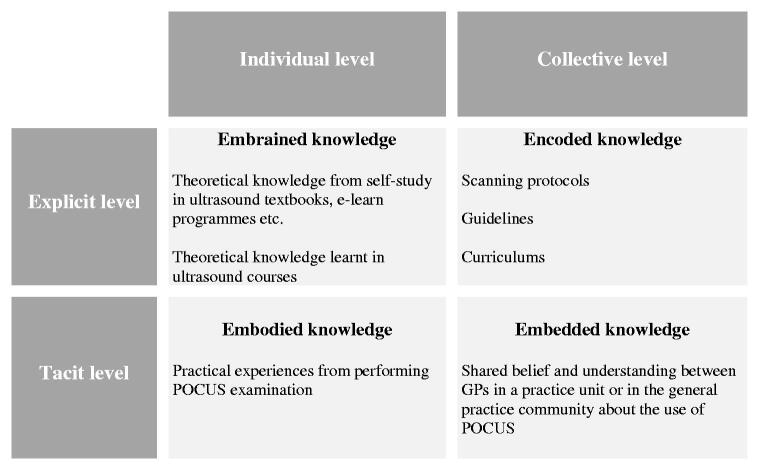
Findings in relation to a model by Lam. The model by Lam [29] describes four types of knowledge. Embrained knowledge can be knowledge learned by reading textbooks or obtained at the theoretical part of an ultrasound course. Embodied knowledge is acquired through individual practical experience and as such it is difficult to transfer from one practitioner to another. Encoded knowledge is codified knowledge stored in procedures, guidelines, curricula etc. to standardize practice; typically provided by central health authorities. Embedded knowledge is collective, tacit knowledge stored in organizational routines based on shared beliefs and understandings in an organization or a community where people collaborate.

At the organisational level, the framework of Lam [26,27] argues that knowledge within a practice unit can reside at an individual and a collective level and can be either explicit or tacit. Figure 4 illustrates our findings in relation to Lam’s four categories of knowledge: Embrained, embodied, encoded, and embedded knowledge. All four kinds of knowledge are relevant for a successful implementation of POCUS. In our study, the GPs described aspects of individual embrained and embodied knowledge. However, they also described the lack of procedural guidance and official recommendations. Furthermore, the large variation in approach and application of POCUS suggests a lack of common understanding in the general practice community. Hence, our findings point to a lack of encoded and embedded knowledge and thereby lack of organisational support to help GPs in their learning process.

Previous studies [12,13] have described how GPs achieve ultrasound proficiency through focused practical training sessions. This study confirms that focused hands-on training is considered an important educational element for GPs striving to master the technical ability to produce ultrasound images. However, other aspects may also be important. The low frequency of POCUS examinations described by the GPs in this study and the low pre-test probability of disease in general practice [16] challenge the ability to learn to recognize pathology on POCUS and to act accordingly. However, this problem was not mentioned by the GPs in this study. The GPs did not describe how they learnt to interpret the POCUS findings or integrate findings in their medical decision-making. In an emergency medicine setting *inability to use results in documentation* was found to be an important barrier for using ultrasound [25]. Whether GPs feel they are sufficiently trained in this aspect we do not know. A recent study comparing the evaluation of left ventricle ejection fraction by expert examiners using standard ultrasound equipment and GPs using a handheld ultrasound device showed that a lack of focus on interpretation of findings in the training programme resulted in poor agreement between expert examiners and GPs [26]. According to the Dreyfus & Dreyfus model [23], including interpretation of findings within the clinical context is important to ensure the GPs’ progression from advanced beginner to competent user.

The GPs in this study described moving through a continuous learning process, which can be translated into the training steps described in guidelines from emergency medicine [1,8]. However, these guidelines include a fixed curriculum and the training steps are therefore not repeated as described by the GPs in this study. In other clinical specialties studies have described how a formal curriculum and a systematic educational programme can increase short- and long-term ultrasound competences [28,29]. In a study of internal medicine residents who had been performing unsupervised POCUS regularly, the authors found that residents had poor understanding of ultrasound. Introducing a longitudinal, multidisciplinary ultrasound curriculum that targeted core ultrasound skills resulted in long-term improvements in the residents’ ultrasound knowledge [29]. These studies support the theory described by Lam [27]; that a successful implementation of POCUS in general practice needs an overall structure.

The GPs in this study had participated in ultrasound courses targeted at secondary care medical specialities. The transition of knowledge from these courses to general practice may be difficult as the patient encounters, prevalence and severity of diseases differ between primary and secondary care [16]. There have been a few suggestions as to which types of examinations should be included in a formal curriculum for teaching POCUS for GPs [3,5,30]. However, as this study has shown, to be successful the curriculum will have to incorporate ultrasound applications that are: (1) relevant in general practice in order to increase the GP’s motivation for investing time and money to learn the skill; (2) regularly encountered in order to ensure that the GPs maintain their ultrasound skills over time; (3) easy to learn to ensure that sufficient competences can be achieved in a short formalized training programme, which minimizes the GP’s absence from the clinic, and (4) not too time-consuming to perform as GPs’ workload is already high and consultation time-slots short.

### Implications for practice and research

Today, POCUS in general practice in Denmark is limited to highly motivated GPs, willing to invest much time and money. However, the use of POCUS in general practice is increasing [10,12]. The variation in training and individual learning strategies for achieving and maintaining POCUS proficiency found in this study suggests the need for more explicit recommendations regarding which POCUS competences GPs should obtain and how they should be obtained before venturing into using POCUS in general practice. This study points to specific learning obstacles for GPs in terms of lack of opportunities for relevant training, practice, supervision and guidance. This may be particularly important as POCUS examinations in general practice are performed rarely and on many different structures. Although, the GPs in this study did not search for scientific evidence to guide them, recommendations should be evidence-based. This study may guide future research examining how GPs are best trained to achieve scanning proficiency, how skills can be maintained over time, and for which scanning modalities obtaining and maintaining competence is feasible in general practice due to especially time constrains and low prevalence of patients with conditions suited for POCUS examinations.
